# Progress in research on the role of fluoride in immune damage

**DOI:** 10.3389/fimmu.2024.1394161

**Published:** 2024-05-14

**Authors:** Siqi Zhu, Wei Wei

**Affiliations:** ^1^ Center for Endemic Disease Control, Chinese Center for Disease Control and Prevention, Harbin Medical University, Harbin, Heilongjiang, China; ^2^ Key Lab of Etiology and Epidemiology, Education Bureau of Heilongjiang Province & Ministry of Health, Harbin Medical University, Harbin, China; ^3^ Heilongjiang Provincial Key Lab of Trace Elements and Human Health Harbin Medical University, Harbin, China

**Keywords:** fluoride, inflammatory response, pathogenesis, immune balance, immune organs, immune cells, immun-active substances

## Abstract

Excessive fluoride intake from residential environments may affect multiple tissues and organs; however, the specific pathogenic mechanisms are unclear. Researchers have recently focused on the damaging effects of fluoride on the immune system. Damage to immune function seriously affects the quality of life of fluoride-exposed populations and increases the incidence of infections and malignant tumors. Probing the mechanism of damage to immune function caused by fluoride helps identify effective drugs and methods to prevent and treat fluorosis and improve people’s living standards in fluorosis-affected areas. Here, the recent literature on the effects of fluoride on the immune system is reviewed, and research on fluoride damage to the immune system is summarized in terms of three perspectives: immune organs, immune cells, and immune-active substances. We reviewed that excessive fluoride can damage immune organs, lead to immune cells dysfunction and interfere with the expression of immune-active substances. This review aimed to provide a potential direction for future fluorosis research from the perspective of fluoride-induced immune function impairment. In order to seek the key regulatory indicators of fluoride on immune homeostasis in the future.

## 1 Introduction

Fluorine is a common non-metallic chemical element that in its elemental form is light yellow gas. Fluorine exists widely in nature primarily as fluorite (CaF_2_), cryolite (Na_3_AlF_6_), and fluorapatite (Ca_5_(PO_4_)_3_F). Fluoride is commonly ingested via drinking water, and excessive intake can affect the development and function of various tissues and organs (1, 2). Fluoridation of drinking water has become a prominent public health problem, and China also has unique health issues stemming from coal-burning fluorosis and tea-drinking fluorosis (3–5). Any form of fluorosis is a serious problem, however, as it can not only cause toxic effects, but also result in cell damage and affect the normal physiological activities of the body. A daily intake of more than 2.4 mg for children aged 8–16 years and 3.5 mg for people aged >16 years may lead to fluorosis and adverse health effects (6). In Ethiopia, India, Tanzania, Mexico, China and other countries, the average fluoride concentration in drinking water is approximately 2 mg/L (7–12). Therefore, the World Health Organization has set the maximum fluoride concentration in water to 1.5 mg/L (13). Long-term ingestion of fluoride causes fluorine to accumulate in the body, which can damage bone tissues and the cardiovascular, nervous, reproductive and digestive systems, as well as result in a range of adverse effects on immune system (14, 15). In recent years, more and more researchers have paid attention to the effect of fluoride on the immune system, founding that fluoride has immunotoxicity, although its pathogenesis is remains unclear. A better understanding of the damage to the immune system by fluorosis is essential.

The immune system plays important roles in immune responses and functions. It may recognize and exclude antigenic foreign bodies, coordinate with other body systems, and maintain the stability of the body’s internal environment and physiological balance (16, 17). The immune system is a special distributed system, that is closely connected with other biological systems, and it is the most effective weapon against pathogen invasion (18, 19). The immune system comprises a network of interactions between immune organs, cells, and active substances, including innate immunity, which provides direct host defense, and adaptive immunity, which has precise memory. Many parameters such as environmental factors and lifestyle can cause immune system abnormalities, leading to the occurrence and development of diseases (20, 21). As the damage caused by fluoride to the immune system may be permanent and systemic, an in-depth understanding of the mechanism of damage to the immune system is of immense importance for the prevention and treatment of endemic fluorosis. In recent years, more and more researchers have studied the immune system damage caused by fluoride and its pathogenesis. Here, we reviewed the effects of drinking water fluorosis on immune system and its potential pathogenesis.

We searched through multiple journal databases (PubMed^@^, CNKI, Google Scholar, etc.) for articles published between 2013 and 2023 in the field of epidemiological investigations and mechanism exploration of fluoride damage to the immune system, and we found that fluoride affects cellular and humoral immunity. Here, the effects of fluoride on immune system in recent years are reviewed in terms of three perspectives: immune organs, immune cells and immune-active substances. This review aimed to provide a potential direction for future fluorosis research from the perspective of fluoride-induced immune function impairment. In light of the non-negligible cumulative damage caused by fluoride to the human body, it is necessary to focus on the changes in tissues and cells at lower doses of fluoride in the future. The results of the authors’ original analysis were used, and no statistical re-analysis was performed.

## 2 Effects of fluoride on immune organs

### 2.1 Bone marrow

Bone marrow is the hematopoietic tissue of the human body. Hematopoietic stem cells in the bone marrow may proliferate and differentiate into erythroid, granuloid, lymphoid, mononuclear, and other blood cells and macrophages (22). Platelets exert hemostatic effects, and white blood cells may kill and inhibit various pathogens, including bacteria and viruses. Some lymphocytes specifically recognize antigens and produce antibodies (23). Therefore, bone marrow is a blood-forming and important immune organ. Owing to its strong affinity for bone, most fluoride accumulates in the calcified tissues of adults, resulting in skeletal fluorosis (24). Therefore, fluoride toxicity in human bones has been widely studied (25). However, the immunotoxic mechanism of fluoride exposure in bone injury remains unclear.

Bone marrow mesenchymal stem cells (BMSCs) are widely used as model cells to evaluate bone toxicity after exposure to environmental toxins. They may self-renew, undergo pluripotent differentiation and maintain immune regulation. Multi-omics analysis combining transcriptomics and metabolomics has shown that the influence of excessive fluoride on BMSCs is primarily caused by the alteration of lysosomes, which are severely damaged by fluoride exposure (26). Osteocytes have a strong tolerance to high fluoride concentrations, while osteoclasts are more sensitive to changes of fluoride dose (25). Fluoride can cause chromosome aberration and micronuclei increase in bone marrow cells, resulting in inhibited proliferation of bone marrow cells (27). In addition, fluoride can not only induce osteoblast differentiation, initiate endoplasmic reticulum stress and unfolded protein response, but also induce genotoxicity, oxidative stress and bone mineralization, thereby affecting cell proliferation and producing inflammatory factors (25, 28–30). However, the limited evidence available suggests that low level of fluoride have no immunosuppressive effect on bone marrow and do not cause immune damage. And low levels of NaF can promote soft tissue healing and hard tissue regeneration (31). Analysis at the transcriptomic level showed that human osteosarcoma (HOS) cells stimulated with sublethal concentration of sodium fluoride (8mg/L) for 30 days significantly changed the expression of genes associated with oxidative stress, inflammation, osteoblast differentiation, and bone development pathways. This suggests that chronic oxidative and inflammatory stress may occur in osteoblasts at sublethal fluoride concentrations, affecting the immune function of bone marrow (27, 32–34). These findings provide new insights to study the mechanism of bone marrow immune damage induced by fluoride.

### 2.2 Thymus

The thymus is an important immune and secretory organ that releases hormones. It is the third line of defense in the human body, providing surveillance and protection and specifically recognizing a variety of pathogens, tumors, antigens, and tissue damage agents. The thymus is the main lymphoid organ involved in T lymphocyte development and regulates T cell development and function. Specific differentiation and generation of T lymphocytes occur in the cortical and medullary regions and are controlled by age (35–37).

Excessive fluoride intake in water may induce immunosuppression, decreasing the number of immune cells and damaging the immune function of the thymus, which can lead to damage via various apoptotic mechanisms and signaling pathways (38). In mature rats, the thymus medulla accounts for 1/3 of the entire lobule, and the ratio of the thymic cortex to the medulla is approximately 2:1 under normal conditions. Fluoride treatment may lead to a decreased ratio of the cortex to the medulla of the thymus, uneven distribution of the cortical layer, irregular ultrastructure and morphology of thymus lymphocytes, and enlargement or cavitation of the mitochondrial crista and apoptotic bodies (39). In addition, fluoride causes enlargement of the thymic nuclear space, condensation, and marginalization of chromatin (40). Moreover, high fluoride levels may decrease the thymus immune index and reduce the number of thymus cells by inhibiting lymphocyte division and promoting cell apoptosis, thereby inhibiting immune function. According to the literature on high fluoride-induced apoptosis, mitochondria-mediated, endoplasmic reticulum (ER)-mediated, and death receptor-mediated pathways are involved (41). However, current studies on the fluoride-induced apoptotic pathways in thymus cells have primarily focused on oxidative stress mediated by the endoplasmic reticulum. Excessive intake of fluoride leads to endoplasmic reticulum stress, which in turn leads to excessive accumulation of the unfolded protein response, activation of RNA activated the protein kinase-like endoplasmic reticulum kinase (PERK), inosin-required protein-1 (IRE1), and activated transcription factor (ATF)-6 signaling pathways. It also triggers eukaryotic initiation factor-2α and tumor necrosis factor (TNF) receptor-associated factor 2 expression. Under continuous external stimulation, ATF4 induces the expression of GADD153, which leads to apoptosis. The immunosuppressant effect of fluoride in the thymus primarily restores cell division reduced by fluoride treatment by inhibiting the PERK and IRE1 signaling pathways, thus inhibiting fluoride-induced apoptosis (42–44).

Fluoride induces the apoptosis of thymus cells and leads to immunosuppression; additionally, high concentrations of fluoride damages thymic epithelial cells (TECs) by altering the expression of T cell function–related genes and the production of immunomodulatory cytokines (45). In TECs, the expression of the *Foxn1*, *Cbx4*, *DLL4*, and *IL-7* genes related to T cell development and differentiation are reduced (46). Thus, a decrease in CD4^+^ and CD8^+^ thymus T cells was induced. The mRNA levels of the immunoregulatory cytokines interleukin-2 (IL-2) and interleukin-10 (IL-10), which are involved in T cell proliferation, were also decreased, and the expression of the T cell function–related genes *CD2*, *PTPRC*, *CD69*, and *CD101* decreased with the action of fluoride. This leads to dysfunction of the thymus gland (47–50). The ingestion of high fluoride levels can hence lead to decreased T cell numbers and T cell dysfunction by reducing the expression of these genes, which may become a new entry point for the study of fluoride-induced immune damage.

### 2.3 Spleen

The spleen is the largest immune organ in the body, accounting for 25% of the total lymphoid tissue. It contains many lymphocytes and macrophages and is the center of cellular and humoral immunity. The spleen’s main function is to filter blood, which also plays an antitumor role via various mechanisms. Removal of the spleen leads to cellular and humoral immunity disorders (51–53).

The immune damage caused by high fluoride levels primarily manifests as a decline in humoral immune function and immune cell apoptosis, thereby reducing the immune response (50). Through the establishment of Wistar rat model of water-induced fluorosis, fluoride was shown to inhibits splenic cell division in rats, decreases the splenic immune index, promotes apoptosis, and leads to the expansion and vesiculation of the endoplasmic reticulum of splenic lymphocytes, with the presence of a large number of vacuoles between cells. Under fluoride treatment, the nuclear space was evident, the chromatin of lymphocytes in the spleen tissue was marginalized, and the nuclear chromatin coagulated (39, 54). In addition, fluoride has been shown to induce endoplasmic reticulum-mediated oxidative stress, activation of PERK and IRE1 signaling, and associated apoptosis. After PERK and IRE1 knockout, cell division ability was restored and apoptosis was reduced in fluoride-treated lymphocytes. This result correlated well with the expression of PERK and IRE1 signaling–related proteins, confirming the key role of these pathways in excessive fluoride immunosuppression. Therefore, the mechanism of fluoride immune damage in the spleen is related to the activation of the PERK and IRE1 signaling pathways. Inhibition of PERK and IRE1 signaling pathways may reduce cell division and apoptotic damage, and high fluoride levels can affect the development and differentiation of immune cells and induce immune cell apoptosis by activating PERK and IRE1 signaling pathways (42, 55–57).

In addition, fluoride may inhibit the activation of the p38/mitogen-activated protein kinase (MAPK) pathway in mouse splenic lymphocytes. *p38/MAPK* mRNA is expressed in macrophages, T lymphocytes, and B lymphocytes in mice. Fluoride may reduce the transcription of *MLK3*, *MKK6*, and *p38/MAPK* genes and thus reduce the expression of MKK6 and p38/MAPK proteins, thereby affecting the immune function of lymphocytes. With an increase in fluoride concentration, the expression of *MKK6*, *p38/MAPK*, and their protein products decreased, which may have been caused by the disruption of homeostasis by chronic fluorosis. These genes are not phosphorylated properly and exhibit differences in gene expression that directly alter protein expression, thereby reducing pathway activity. The p38/MAPK pathway is activated, and the c-Jun N-terminal kinase (JNK) and extracellular regulated protein kinases (ERK) pathways may also be activated, and high fluoride concentrations have a substantial inhibitory effect on mouse B lymphocytes, reducing the proliferative activity of lymphocytes, indicating that sodium fluoride (NaF) may inhibit the proliferation and differentiation of lymphocytes via the p38/MAPK pathway (58–61).

The damage of fluoride to the spleen is not only manifested in morphology and apoptosis but also leads to an increase in the percentage of G0/G1 phase cells and a decrease in the number of S phase cells in the spleen (62). Cell cycle arrest is the molecular basis for NaF toxicity caused by NaF during spleen development. Moreover, the protein expression levels of IL-2, transforming growth factor-β (TGF-β), TNF-α, interferon-γ (IFN-γ), and cyclin E/D and CDK2/4 were significantly decreased. In contrast, the IL-10 protein expression level was significantly increased. The expression levels of cyclin E/CDK2 and cyclin D/CDK4 proteins were significantly reduced, indicating that NaF treatment slowed the G1 cell cycle progression, blocked the G1/S transition, inhibited splenic lymphocyte proliferation, and damaged DNA synthesis, thereby resulting in cytotoxicity in mice (49, 63–66).

### 2.4 Intestinal mucosal and intestinal lymph node

The intestine is an important digestive organ in the human body by playing key roles in food digestion and absorption, water and electrolyte balance, and immune regulation. The intestines can absorb the necessary nutrients in food, regulate the osmotic pressure of plasma crystals, and monitor and eliminate pathogenic bacteria via specific and non-specific immunity to protect the body’s internal environment (67). People living in fluoride-endemic areas often report stomach abnormalities with symptoms including loss of appetite, nausea, anorexia, gas, constipation, and intermittent diarrhea (68). Fluoride is absorbed in the gastrointestinal tract, and numerous studies in laboratory animals and humans have shown that ingestion of NaF may cause gastrointestinal damage (69–71). In addition, long-term excessive intake of fluoride has a certain damaging effect on the intestine, inhibiting the proliferation of epithelial cells (72), leading to increased intestinal permeability, and thus destroying the mechanical and immune barrier function of the intestine (73).

Dietary high fluoride–induced oxidative damage decreased the percentage of T cell subgroups in the cecal tonsil and decreased Immunoglobulin A (IgA), Immunoglobulin G (IgG), and Immunoglobulin M (IgM) levels in the cecal tonsil, which ultimately affected the local mucosal immune function of broilers (71, 74). Morever, ingested fluoride is absorbed from the stomach and intestinal epithelium. Excess fluoride may inhibit intestinal epithelial cell and mast cell proliferation, promote the release of bioactive molecules, stimulate inflammatory cells, activate lymphocytes, reduce the concentrations of immunoglobulins, reduce the ability to resist pathogen infection, and affect the immune function of the intestinal mucosal (69, 75–78). The intestinal barrier is considered to be the most important defense against microbial pathogens that enter the host through the gut (79). Excessive fluoride can stimulate the expression of pro-inflammatory factors, reduce the expression level of tight-junction related genes and proteins, activate inflammatory response, promote cell pyrogen, inhibit intestinal development, and thus cause intestinal inflammation and diarrhea (80). In addition, using high-throughput 16S rRNA gene sequencing technology, it has been found that fungi may be one of the important factors affecting intestinal function, and excessive fluorine can affect the species richness of intestinal fungi in mice, suggesting that fluorine may cause adverse intestinal symptoms by causing an imbalance in the intestinal fungal population, thus affecting the normal intestinal function (81, 82). Moreover, fluoride exposure not only disturbed the compositional balance of the gut microbiome, but also triggered metabolic disorders (83). Alterations in intestinal microbiota and metabolome play a critical role in regulating disease susceptibility and multi-organ damage after excessive fluoride exposure (73). More and more studies have shown that there is a significant difference in the abundance of bacteria in the gut after excessive fluoride intake, and excessive fluoride changes the composition of intestinal microbes in animals (77, 84, 85).

## 3 Effects of fluoride on immune cells

The innate immune system is the body’s second line of defense. The cells involved in innate immune response are primarily phagocytes, including macrophages, neutrophils, and natural killer (NK) cells. The adaptive immune system is activated after the body comes in contact with foreign microorganisms after birth. Adaptive immunity includes cellular and humoral immunity, and the cells involved in the adaptive immune response are primarily lymphocytes, including T lymphocytes and B lymphocytes (86–89).

### 3.1 Macrophages

Macrophages are an important part of the innate immune system, and their activation is indispensable for several aspects of immune defense, inflammatory responses, tissue remodeling, and homeostasis. Macrophages are found in almost all body tissues and are highly heterogeneous. Macrophages function in different physiological states primarily via cell polarization (90).

High fluoride stimulation may affect macrophage polarization, resulting in decreased M2 differentiation and inhibition of osteogenic differentiation and formation (91). Moreover, macrophages exposed to fluoride concentrations ≥50 mg/L (2.6 mM NaF) produce active substances, significantly increasing lipid peroxidation. Even within a short period, it leads to macrophage redox imbalance, resulting in a decline in mitochondrial activity and phagocyte vitality (49). Additionally low fluoride levels can also damage the immune system. Even 1 and 3μM NaF can also affect the expression and activity of 15 lipoxygenases, the number and activity of cyclo-oxygenase, and the activation of phospholipase A2 in human peripheral blood mononuclear cell differentiation mononuclear cells, and promote the biosynthesis of inflammatory mediators, leading to inflammation (92, 93). Macrophages are the main source of reactive oxygen species (ROS) in the human body, participate in the body’s response to various pathogenic factors, and play a key role in inflammation. Under the effect of high fluoride concentrations, the formation of ROS in macrophages increases oxidative stress, thus affecting the expression of inflammatory factors (94).

### 3.2 Neutrophils

Neutrophils are effective antibacterial cells, capable of attacking microorganisms within the limited intracellular compartment with powerful antibacterial properties (95). Inappropriate or dysregulated neutrophil activation may damage the host and lead to autoimmune and inflammatory diseases (96).

Due to the harmful effects of fluoride on the bone marrow and hematopoietic organs, fluoride may lower white blood cell, platelet, and neutrophil counts (97). Fluoride stimulation of neutrophils isolated from the tail venous blood of healthy carp showed that NaF significantly increased the release of neutrophil extracellular traps (NET) by regulating the Adenosine 5’-monophosphate (AMP)-activated protein kinase/p38 (AMPK/p38) signaling pathway, suggesting that the toxic effect of fluoride on the immune system of carp may be caused by the release of NET (98). In addition, NaF-induced NET formation in bovine neutrophils is accompanied by increased ROS production and decreased antioxidant enzyme activity, and oxidative stress and NET formation may cause immunotoxicity in neutrophils via the p38/MAPK and ERK pathways (99).

### 3.3 NK cells

NK cells are lymphocytes of the innate immune system that play an important role in innate and adaptive immune responses to tumors and viral infections. They not only exert cell-mediated cytotoxicity toward tumor and infected cells but also play a regulatory role by secreting cytokines and chemokines to promote or inhibit the functions of other immune cells (100, 101).

NaF has immunosuppressive activity, and fluoride may enhance the *in vitro* reactivity of human lymphocytes to mitogen or specific antigens. Increased levels of IFN-γ and sIL-2R, which are products of activated T and NK cells, suggest that NaF may primarily act on T and/or NK cells (102). The levels of primary immune response cells, namely CD4 cells, IgG1 cells, and NK cells, may be reduced by different fluoride concentrations. Lipid peroxidation increases significantly, and the antioxidant defense system is damaged (103).

### 3.4 T lymphocytes

T lymphocytes are derived from pluripotent stem cells in the bone marrow. During the embryonic and neonatal stages, some pluripotent stem cells or pro-T cells in the bone marrow migrate to the thymus and mature into immunoactive T cells under the induction of thymus hormones (104–106). T lymphocytes are divided into two functionally distinct subsets: CD4^+^ T helper (Th) cells and CD8^+^ cytotoxic T lymphocytes (CTL). These cells are indirectly involved in clearing infections by regulating the activities of other immune cells. CTL cells induce apoptosis by secreting pro-inflammatory cytokines. Adequate CD4^+^ and CD8^+^ T cell activation, proliferation, clonal amplification, and effector functions are essential to clear the infection effectively (107, 108).

Exposure to excessive fluoride reduces the number of lymphocytes, resulting in a decrease in the percentage of CD4^+^ and CD8^+^ T cells in the thymus, leading to T cell dysfunction (109). Excessive fluoride intake can alter T-bet and GATA3 protein expression levels in the spleen. T-bet and GATA3 are transcription factors that effectively regulate the development, differentiation, and memory formation of Th1/Th2 cells, disrupting the balance of Th1/Th2 cells (110). Splenic injury is eventually induced by alterations in the expression of Th1/Th2 cell–related cytokines in the splenic microenvironment. In addition, excessive fluoride intake has been found to affect the number of Th17 cells and the expression of related cytokines, leading to changes in the immune system (111–113).

### 3.5 B lymphocytes

B lymphocytes are derived from the mammalian bone marrow or bird bursa lymphoid stem cells, also called bone marrow- or sac-dependent lymphocytes. They primarily settles in the superficial cortical area of the lymph nodes and lymph nodules of the white pulp of the spleen. In peripheral blood, B cells account for approximately 10%-15% of lymphocytes (113). B cells are the main cells involved in humoral immunity and produce immune effects primarily by secreting antibodies (114–117).

A study using flow cytometry to determine the percentages of CD3^+^, CD3^+^CD4^+^, CD3^+^CD8^+^ T, and CD19^+^ B lymphocytes in the spleens of mice (118). The results showed that the percentage of B lymphocytes was significantly reduced under high fluoride conditions. In addition, a high fluoride diet reduced the number of tonsil B lymphocytes in the cecum and B lymphocytes in the intestinal mucosal of broilers (71, 74, 119). The decrease in the number and activity of B lymphocytes may be caused by the apoptosis of B lymphocytes induced by NaF or the inhibition of lymphocyte proliferation. Some studies have shown that NaF may induce apoptosis and inhibit the proliferation of splenic B lymphocytes both *in vivo* and *in vitro*, thus impairing humoral immunity (120).

## 4 Effects of fluoride on immun-active substance

Immun-active substances are produced by immune cells and other cells involved in immune responses for resisting or preventing infection by microorganisms or parasites or invasion by other organisms. They primarily comprise immunoglobulins, interferons, interleukins, TNFs, and other cytokines.

Immunoglobulins are used to assess the immune system (121). The downregulation of IgA, IgG and IgM is mainly due to the decrease in the number of B lymphocytes and the inhibition of Th2 cytokine expression. Fluoride reduces the number of B lymphocytes, which in turn leads to decreased expression of IgA, IgG, and IgM in serum (122, 123). In conclusion, NaF can reduce lymphocyte proliferation and inhibit humoral immune function.

Cytokines are a class of small molecular proteins that are synthesized and secreted by immune cells and some non-immune cells in response to stimulation and have a wide range of biological activities, including the regulation of innate and adaptive immunity, hematogenesis, cell growth, and repair of damaged tissues. Cytokines include interleukins, interferons, the TNF superfamily, colony-stimulating factors, chemokines, and growth factors. Th1 cells produce IL-2, IFN-γ, and TNF, which are involved in cellular immunity. Th2 cells produce IL-4, IL-6, and IL-10 and participate in humoral immunity. Th17 cells produce pro-inflammatory cytokines such as IL-17, which are important for inducing inflammation (124). NaF may reduce serum Th1 cytokines (IL-2, IFN-γ and TNF) and Th2 cytokines (IL-4, IL-6 and IL-10) and lead to a decrease in the number of CD3^+^CD4^+^ T cells (125). It has also been found that Th17 cells primarily produce IL-17 (126). Th17 cells may eliminate pathogens inadequately eliminated by Th1 or Th2 cells. The results showed that IL-17A levels were significantly decreased in the cecum and increased in the rectum after exposure to NaF (127). At the same time, fluoride exposure significantly up-regulated the expression of intestinal IL-1β, IL-23 and IL-22, while IL-6 levels were down-regulated (72). Morever, high fluoride levels may reduce IL-2, IL-4, IL-6, TNF-α, and IFN-γ levels in broilers’ cecal tonsil and intestinal tract (128, 129). High fluoride levels may decrease the percentage of CD4^+^ cells and the mRNA expression of IL-2 and IL-10 in the thymus (130). In addition, NaF has been reduced the expression of IL-10 mRNA in mouse macrophages, and the decrease in cytokine levels indicates that excessive intake of NaF may inhibit cellular immunity and immune function in mice. In Pakistan, pro-inflammatory cytokines (TNF-α, IL-1β, and IL-6) were increased in people who drank high fluoride-contained water over a long period of time, and there was a significant correlation between fluoride exposure and cholinergase. This may be due to low-grade systemic inflammation through the cholinergic pathway (131). Besides, NaF significantly increases the mRNA and protein levels of MAPK signaling pathways and significantly inhibits the production of pro-inflammatory mediators such as IL-1β, IL-6, IL-8, monocyte chemotactic protein 1, and cyclooxygenase-2 and anti-inflammatory mediators, including IL-4 and TGF-β, thus resulting in the body’s immune dysfunction and inflammatory response (132–134).

## 5 Conclusion

An increasing number of researchers have focused on the damage to the immune system caused by endemic fluorosis, and substantial progress has been made in understanding the mechanism of immune system damage. Recent studies have shown that fluoride damages the immune system primarily via oxidative stress pathways, signaling pathways, cell apoptosis, and cell activity. For example, several studies have found that fluoride regulates cell differentiation and development primarily via the oxidative stress pathway when it damages immune organs such as the thymus and spleen, thus leading to cell apoptosis and inhibition of relevant signaling pathways. Therefore, immune system damage warrants further exploration. These findings provide a new direction for studying other fluoride-induced immune dysfunctions and lay the foundation for subsequent studies at the molecular level.

**Figure 1:**
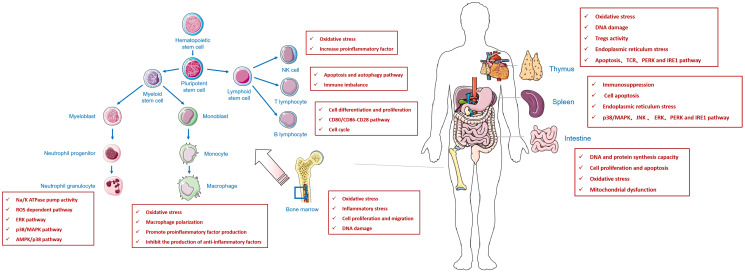
An review of the effect of fluoride on immune system.

In this review, we found that fluoride affects both cellular and humoral immunity. Fluoride decreases the number and activity of immune cells, causing an imbalance in the Th1/Th2 cells. However, an imbalance in the Th1/Th2 cell ratio may lead to a response from the body’s immune system to its components, leading to autoimmune diseases. In contrast, T lymphocyte subsets in peripheral blood are changed, the number of auxiliary T lymphocytes is reduced and the number of inhibitory T lymphocytes is relatively high, leading to tumor immunosuppression, which affects the occurrence and development of tumors (**Figure 1**).

In addition to further exploring the molecular mechanisms underlying the relationship between fluoride and immune organs, immune cells, and immune-active substances, future studies on fluoride-induced immune damage should also explore the relevant immune homeostasis, stress response, immunotherapy, and signal transduction pathways regulated by fluoride. Second, multi-omics and epigenetic technologies, such as transcriptomics and proteomics, are used to find a breakthrough conducive to a deeper understanding of whether fluoride-induced body damage affects the occurrence and development of tumors in different genetic backgrounds. Can fluoride be used as an immune adjuvant in the development of autoimmune diseases and cancer? In addition, studies have found that more and more diseases can be treated with immune cells to suppress the immune response, thus reducing the occurrence of diseases. It is estimated that approximately 100 million people worldwide have fluoride levels in their drinking water above the maximum safe level of fluoride specified by the World Health Organization (WHO) regulations (135). Can immune cell therapy treat patients with fluorosis and improve their quality of life? Finally, several studies to date have focused on the irreversible damage to the immune system caused by high fluoride concentrations (**Tables 1**–**3**), mostly at concentrations unavailable to humans in nature. The most recent standard for drinking water in China has a limit of 1.2mg/L for fluoride, and different types of fluorosis persist in China (139). The United States requires that the final concentration of fluoride added to bottled water does not exceed 0.7mg/L (140), and Taiwan requires that the concentration of fluoride in drinking water does not exceed 0.8mg/L (141). Considering the non-negligible cumulative damage caused by fluoride to the human body, it is necessary to focus on the changes in tissues and cells at lower doses of fluoride in the future.

**Table 1:** Effects of fluoride on immune organs.

Immune Organs	Fluoride Concentration	Subjects	Outcome	Mechanism	Reference
Bone marrow	8mg/L	Human osteosarcoma (HOS) cells	• Upregulation: RARRES3, SAA1, IFITM1, APOL1, CCL2, ANXA8 • Downregulation: SMAD5, WWTR1, TRAM2, SATB2, HIF1A, CLTC, RUNX2, RANKL,OPG	• Oxidative stress • Inflammatory stress • Osteoblastic differentiation • Bone development pathway	32
	50, 500 μM and 5 mM	Bone marrow mesenchymal stem cells (BMMSCs)	• Upregulate osteogenic differentiation • Promote soft tissue healing • Hard tissue regeneration	• Cell proliferation and migration • Osteogenic differentiation	31
	25.1 mg/kg body weight (BW)/d	Swiss albino mice bone marrow cells	• Increased chromosomal aberration and micronuclei • Produce reactive oxygen species • Promote genetic instability	• Oxidative stress • DNA damage • Genotoxicity	27
	4, 12 and 20 mg/L	Adult Swiss albino male mice	• DNA strand breaks • Structural chromosome aberrations	• DNA damage • Genotoxicity	136
	0.1, 0.2, 0.4, 0.8 and 1.6 mM	Human osteoblast-like cell line(Saos-2)	• Promotes osteoblastic differentiation • Major influence concentration: 0.2 mM • Phosphorylated Smad1/5 increased	• BMP/Smad pathway	28
Thymus	20 mg/kg body weight (BW)/d	Male albinoWistar rats	• Catalase and peroxidase activity decreased • Obstruction tissue structure disorder	• Oxidative stress	39
	150mg/L	Female Wistar rats	• Change lymphocytes ultrastructure • Upregulate caspase-3 and caspase-9	• Apoptosis pathway • DNA damage	40 41
	50,100 and 150 mg/L	Female C57BL/6 strain mice	• Decrease T cell development and differentiation • Reduce cytokine expression	• TCR pathway • Tregs activity	45 44
	50 and 100 mg/L	Male Wistar rats	• Thymus atrophies • Inhibit cell division • Upregulate caspase-3 and caspase-12	• PERK and IRE1 pathways • Endoplasmic reticulum stress	38
Spleen	50,100 and 150 mg/L	Male KM mice	• Inhibit cell proliferation • Induce apoptosis	• p38/MAPK singaling pathway • JNK,ERK singaling pathway	59
	12, 24 and 48 mg/kg	ICR mice	• Block cell cycle • Change histopathological • Reduce immunoglobulin level	• Cell apoptosis • Endoplasmic reticulum stress	42
	100 mg/L	Wistar rats (female:male=3:1)	• Splenic lymphocyte proliferation decreased • Block cell cycle • Inhibit spleen development	• ERK pathway	43
	50 and 100 mg/L	Male Wistar rats	• Inhibit cell division • Promote apoptosis	• PERK and IRE1 pathways • Immunosuppression	56
	20 mg/kg body weight (BW)/d	Male albinoWistar rats	• Catalase and peroxidase activity decreased • Obstruction tissue structure disorder • Splenocyte counts decreased • Malondialdahyde increased	• Oxidative stress	39
Intestinal mucosal and intestinal lymph node	400, 800 and 1200 mg/kg	Broilers	• Promote cell proliferation • Induce Bcl-2, Bax and Caspase-3 • Impact immune defending in the early-stage	• Cell apoptosis • DNA and protein synthesis capacity • Cell proliferation	75
	25, 50 and 100 mg/L	Rat model of estrogen deficiency	• Inflammatory cytokines increased • Goblet cells and glycoproteins decreased	• Cell proliferation • Mucus barrier	72
	50, 100 and 125 mg/L	Male C57BL/6 mice	• Secondary red blood cell immune dysfunction • Reduce neutrophils • Decreased NK cell activity • Inflammatory cytokines increased	• Number and activity of immune cells	73
	25mg/kg	Female Sprague Dawley rats	• Villi propria lymphocyte excess, chorioedema, focal ileitis, villi necrosis and ulceration	• Oxidative stress • Mitochondrial dysfunction.	76

**Table 2:** Effects of fluoride on immune cells.

Immune Cells	Fluoride Concentration	Subjects	Outcome	Mechanism	Reference
Macrophage	5, 10, 20 and 50 mg/L	RAW 264.7 cells	• Reduce macrophage population • Increase lipid peroxidation	• Pro-inflammatory cytokines • Anti-inflammatory cytokines	49
	3 μM	THP-1 macrophage	• Increase ROS formation • Promote inflammatory factors	• Oxidative stress	91
	5.0 and 10 mM	Male Sprague–Dawley rats	• Inhibit the osteogenesis and angiogenesis differentiation of MSCs • Decrease cytokine expression	• Macrophage polarization	90
Neutrophil	100mg/L	Male Wistar rats	• Difference in hematological parameters	• Na/K ATPase pump activity	96
	0.25, 0.5 and 1 mM	Bovine neutrophils	• Increased ROS production • Decreased antioxidant enzyme activity	• ROS dependent pathway • ERK pathway • p38/MAPK pathway	98
	0.25, 0.5 and 1 mM	Crap neutrophils	• Induce the formation of NETs • Promeote ROS production	• AMPK/p38 pathway	97
	50, 100 and 125 mg/L	Male C57BL/6 mice	• Secondary red blood cell immune dysfunction • Reduce neutrophils • Inflammatory cytokines increased	• Number and activity of immune cells	73
	20 mg/kg body weight (BW)/d	Male albinoWistar rats	• Neutrophils counts decreased	• Oxidative stress	39
NK cells	2.5 mmol/L	Peripheral blood mononuclear cells	• Increased IFN-γ production	• IFN-γ, IL-2 receptor	100
	0.8 (control), 20, 60 and 100 ppm	Male Wistar rats	• Decrease immene cells • Impaire antioxidant defense systems • Increase lipid peroxidation	• Oxidative stress	102
	50, 100 and 125 mg/L	Male C57BL/6 mice	• Secondary red blood cell immune dysfunction • Decreased NK cell activity • Inflammatory cytokines increased	• Number and activity of immune cells	73
T Lymphocyte	25, 50, 100 mg/L	Kunming male mice	• T lymphocyte activation • Hepatic immunotoxicity	• Apoptosis pathway • Autophagy pathway • IL-17 signaling pathway	112
	25, 50 and 100 mg/L	Kunming male mice	• Splenic immunotoxicity • Inhibit Th1 activity and promote Th2 • Decrease TGF-β,IFN -γ,IL-2,IL-4,IL-6 and IL-10	• Th1/Th2 immune imbalance	109 110
B Lymphocyte	50, 100, 500 and 1000 µM	Lymphocyte from spleen of Kunming female mice	• Enhance the endocytosis of B cells • Reduce the number of B cells	• Plasma cell differentiation • CD80/CD86-CD28 pathway • CD40-L/CD154	118
	100, 500 and 1000 μmol/L	Male ICR mice	• Cell cycle arrest • Reduce IL-2, TNF-α, IFN-γ and TGF-β • Inhibit lymphocyte proliferation	• Cell cycle • Cell proliferation	120

**Table 3:** Effects of fluoride on immune-active substances.

Immune-active substances	Fluoride Concentration	Subjects	Outcome	Mechanism	Reference
Immunoglobulin	12, 24 and 48 mg/kg	ICR mice	• IgA, IgG and IgM decreased	• Cell cycle arrest • DNA synthesis • Cell proliferation	122
	50, 100 and 125 mg/L	C57BL/6 mice	• The level of IgA increased first and then decreased	• Immune cell function decline • Cell apoptosis	73
	400, 800 and 1200 mg/kg	Broilers	• IgA, IgG and IgM decreased	• Mucosal immune	71, 80
	20 mg/kg body weight (BW)/d	Male albinoWistar rats	• IgG decreased	• Oxidative stress	39
Cytokine	50, 100 and 125 mg/L	C57BL/6 mice	• IL-1β and IL-8 increase	• Local inflammatory responses • Intestinal immune barrier	73
	12, 24 and 48 mg/kg	ICR mice	• IL-2, TNF-α, TGF-β and IFN-γ decreased • IL-10 increased	• Cell cycle arrest • DNA synthesis • Cell proliferation	122
	50 and 100 mg/L	C57 mice	• IL-17A increased	• IL-17A pathway • Mitophagy	127
	25, 50, and 100 mg/L	Female Sprague-Dawley rats	• IL-1β, IL-2, IL-6, and TNF-α decreased	• Mucosal immune • Intestinal development	137
	12, 24, and 48 mg/kg	Mice	• IL-1β, IL-6, IL-8, MCP-1 and COX-2 increased • IL-4 and TGF-β decreased	• MAPKs/NF-κB • Cell apoptosis	33
	25, 50 and 100 mg/L	Rat model of estrogen deficiency	• Increase IL-1β, IL-6, IL-23, and IL-22 • Decrease IL-17A	• Cell proliferation • Mucus barrier	72
	fluoride, exercise group	Male ICR mice	• IL-1β, IL-2, IL-4, IL-6, IL-10, IL-12, IL-13, IL-21, TNF-α and TGF-β increased	• IKKβ/NFκB pathway • Inflammatory balance	138

## Author contributions

SZ: Conceptualization, Formal Analysis, Investigation, Methodology, Validation, Writing – original draft. WW: Funding acquisition, Project administration, Resources, Supervision, Writing – review & editing.
